# Public health round-up

**DOI:** 10.2471/BLT.24.010724

**Published:** 2024-07-01

**Dengue surges**

A mother and child await to be admitted to the dengue ward at Mugda Hospital in Dhaka, Bangladesh, one of many countries to be experiencing a sharp increase in dengue cases. As of 30 April 2024, over 7.6 million dengue cases had been reported to WHO worldwide.

**Figure:**
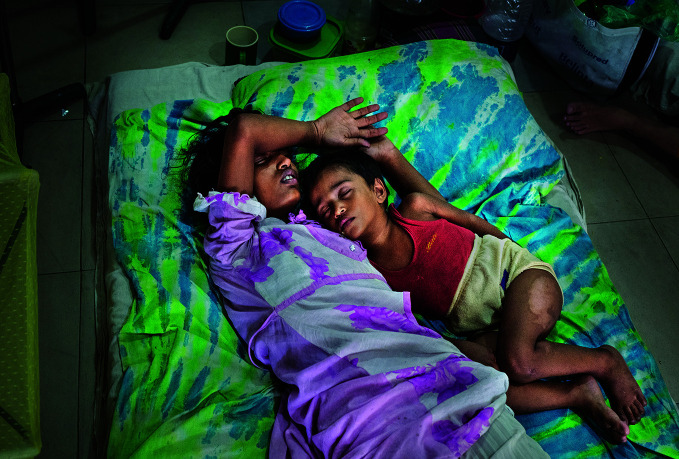
WHO/Billy Miaron

## Gaza crisis

The United Nations (UN) Security Council, on 10 June, adopted a resolution aimed at reaching a comprehensive, three-phase ceasefire deal to end the war in Gaza. At a 12 June World Health Organization (WHO) media briefing, Director-General Tedros Adhanom Ghebreyesus urged all parties to implement the resolution.

Drawing particular attention to the development of what he described as “famine-like” conditions in Gaza, the Director-General reported that malnutrition had already caused 32 deaths, including the deaths of 28 children. WHO has scaled up nutrition services in Gaza, diagnosing and treating over 8000 children for acute malnutrition as of 12 June.

The Director-General also drew attention to the situation in the West Bank, where the escalating health crisis is being exacerbated by attacks on health care and restrictions on movement of people.

https://bit.ly/4cfAwao

https://bit.ly/4cikZXr

## Sudan famine

Three UN agencies issued a warning regarding a significant deterioration of the nutrition situation for children and mothers in war-torn Sudan. Issued on 30 May, the warning is based on a recent analysis by the United Nations Children’s Fund, the World Food Programme and WHO, which describes ways in which the war is impacting access to nutrition, safe drinking water and sanitation, with serious implications for increased risk of disease.

Massive population displacement and disrupted humanitarian aid delivery have compounded the crisis, pushing Sudan towards a conflict-induced famine with potentially catastrophic consequences for young children. The agencies called for immediate action to prevent further deterioration and protect the lives of Sudan's vulnerable population.

The WHO Director-General also drew attention to the crisis in his 12 June media briefing, pointing out that Sudan is undergoing the world’s largest humanitarian crisis.

https://bit.ly/3yyphv5

https://bit.ly/3Vweued

## Infectious disease trends

Sexually transmitted infections (STIs) are on the rise in many regions, according to a new WHO report, *Implementing the Global Health Sector Strategies on HIV, Viral Hepatitis, and Sexually Transmitted Infections, 2022–2030*, published on 21 May.

The first biannual progress report reveals significant challenges and slow progress toward key health targets. Annual deaths from these diseases remain at 2.5 million, with hepatitis-related deaths increasing from 1.1 million in 2019 to 1.3 million in 2022. Over 1 million new infections occur daily, predominantly STIs, with cases rising in several WHO regions.

The report calls for an urgent acceleration of global health efforts to meet the 2025 and 2030 targets.

https://bit.ly/3WQASzZ

## Dengue cases up sharply

There was a three-fold year-on-year surge in dengue cases in the Region of the Americas in the first four months of 2024. According to the 30 May issue of Disease Outbreak News, there had been more than seven million reported cases in the region by the end of April 2024, tripling the number reported during the same period in 2023, and surpassing the 4.6 million full-year figure for 2023.

As of 30 April 2024, over 7.6 million dengue cases had been reported to WHO including over 16 000 severe cases, and over 3000 deaths.

WHO has established a global dengue surveillance system with monthly reporting across all regions, to strengthen surveillance, monitor disease incidence and provide support to high-risk countries across affected regions.

https://bit.ly/45q5hHG

## Amended international health regulations

The Seventy-seventh World Health Assembly (WHA77) passed amendments to the* International health regulations* (2005) (IHR) and committed to finalizing a global pandemic agreement within a year.

The amendments focus on enhancing global preparedness, surveillance and response to public health emergencies. Key changes include establishing a definition for a pandemic emergency to improve international response and collaboration, and strengthening access to medical products through the creation of a coordinating financial mechanism. 

In a 1 June media release, the WHO Director-General said the actions taken by the Assembly reflected a common desire by Member States to protect their own people and the world from the shared risk of public health emergencies and future pandemics.

https://bit.ly/3VknXDZ

## Resolution on climate and health

WHA77 passed a landmark resolution recognizing climate change as an imminent threat to global health. Backed by overwhelming support from Member States, the resolution highlights the critical need for urgent action to address the profound health risks posed by climate change.

WHO remains committed to leading the global health response to climate change, scaling up efforts to support Member States through leadership, awareness-raising, and capacity-building, while advocating for health-centric climate policies and evidence-based strategies.

https://bit.ly/3XrOq4W

## WHO strategy approved

Delegates at WHA77 approved the Organization’s Fourteenth General Programme of Work (GPW 14). A four-year strategy for global health to promote, provide and protect health and well-being for all people, GPW 14 puts the emphasis on climate change, aging, migration, pandemic threats and equity, and reflects the need to adapt to fast-moving science and technology.

According to a 28 May media release, the strategy targets 2025–2028 as a critical period in which to recover from the COVID-19 pandemic, get back on track to reach the health-related sustainable development goals, and to build resilient, fit-for-future health systems.

https://bit.ly/3V25VpP

## Commercial determinants report

Tobacco, ultra-processed foods, fossil fuels and alcohol cause 2.7 million deaths annually in the European Region. A report produced by the WHO Regional Office for Europe describes how four industries are driving ill-health and premature deaths across Europe and central Asia.

Launched on 12 June by Belgian Deputy Prime Minister and Minister of Social Affairs and Public Health, Frank Vandenbroucke, in partnership with the WHO European Forum on Commercial Determinants of Noncommunicable Diseases, the report sheds light on the wide range of tactics industries employ to maximize profits and undermine public health, and identifies actions for governments, academia and civil society to reduce the disproportionate influence of the commercial sector in the health policy sphere.

https://bit.ly/3KEflmE

**Cover photo**

A mobile health clinic in Marawi City, southern Philippines.

**Figure:**
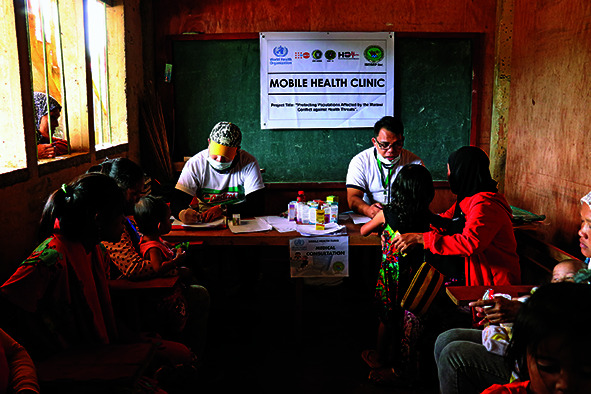
(WHO/Faizza Tanggol)

## Updated bacterial priority pathogens list

WHO released its updated Bacterial Priority Pathogens List (BPPL) 2024, identifying 15 families of antibiotic-resistant bacteria categorized as critical, high, or medium priority.

Published 17 May, the updated BPPL incorporates new evidence and expert insights to guide research, foster innovation and promote international coordination.

“This list is key to guiding investment and grappling with the antibiotics pipeline and access crisis," said Dr Yukiko Nakatani, WHO’s interim Assistant Director-General for Antimicrobial Resistance.

https://bit.ly/4elWwCa

## Mpox prevention and control

WHO released a framework for the prevention and control of mpox on 24 May. The new framework is designed to guide health authorities, communities and stakeholders in preventing mpox outbreaks, eliminating human-to-human transmission and reducing spillover from animals to humans.

A major emergence of clade II mpox (one of two mpox clades or groups) began in 2017, and since 2022 has spread to all WHO regions, with reports suggesting that low-level transmission continues worldwide. A major outbreak of clade I mpox virus in the Democratic Republic of the Congo is ongoing, with over 6500 cases and 345 deaths reported since the beginning of the year.

https://bit.ly/4b1VmZY

**Looking ahead**

24-25 July, Global Public Health Conference 2024. Paris, France. https://globalpublichealth.healthconferences.org/

28 July, World Hepatitis Day. https://www.worldhepatitisday.org/

16-17 August, Machine Learning for Healthcare. University of Toronto, Canada. https://www.mlforhc.org/

